# Propan-1-aminium 3,4,5,6-tetra­bromo-2-(meth­oxy­carbon­yl)benzoate *N*,*N*-dimethyl­formamide monosolvate

**DOI:** 10.1107/S1600536811016850

**Published:** 2011-05-07

**Authors:** Jian Li

**Affiliations:** a Department of Chemistry and Chemical Engineering, Weifang University, Weifang 261061, People’s Republic of China

## Abstract

In the anion of the title solvated molecular salt, C_3_H_10_N^+^·C_9_H_3_Br_4_O_4_^−^·C_3_H_7_NO, the dihedral angles formed by the aromatic ring and the mean planes of the carboxyl­ate and meth­oxy­carbonyl groups are 64.3 (3) and 75.2 (3)°, respectively. The C atoms of the propan-1-aminium cation are disordered over two sets of sites in a 0.65 (3):0.35 (3) ratio. The crystal structure is stabilized by N—H⋯O hydrogen bonds.

## Related literature

For related structures, see: Li (2011*a* ▶,*b* ▶).
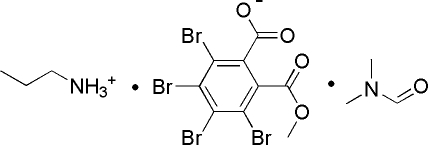


## Experimental

### 

#### Crystal data

C_3_H_10_N^+^·C_9_H_3_Br_4_O_4_^−^·C_3_H_7_NO
*M* *_r_* = 627.97
Monoclinic, 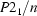

*a* = 11.8964 (11) Å
*b* = 10.5198 (10) Å
*c* = 17.3743 (17) Å
β = 93.188 (1)°
*V* = 2171.0 (4) Å^3^
*Z* = 4
Mo *K*α radiation
μ = 7.44 mm^−1^
*T* = 298 K
0.40 × 0.37 × 0.24 mm

#### Data collection

Bruker SMART CCD diffractometer
Absorption correction: multi-scan (*SADABS*; Bruker, 1997 ▶) *T*_min_ = 0.155, *T*_max_ = 0.268
10672 measured reflections
3821 independent reflections
1836 reflections with *I* > 2σ(*I*)
*R*_int_ = 0.069

#### Refinement

*R*[*F* ^2^ > 2σ(*F* ^2^)] = 0.047
*wR*(*F* ^2^) = 0.104
*S* = 1.03
3821 reflections
268 parameters
H-atom parameters constrained
Δρ_max_ = 0.57 e Å^−3^
Δρ_min_ = −0.38 e Å^−3^

### 

Data collection: *SMART* (Bruker, 1997 ▶); cell refinement: *SAINT* (Bruker, 1997 ▶); data reduction: *SAINT*; program(s) used to solve structure: *SHELXS97* (Sheldrick, 2008 ▶); program(s) used to refine structure: *SHELXL97* (Sheldrick, 2008 ▶); molecular graphics: *SHELXTL* (Sheldrick, 2008 ▶); software used to prepare material for publication: *SHELXTL*.

## Supplementary Material

Crystal structure: contains datablocks global, I. DOI: 10.1107/S1600536811016850/bt5538sup1.cif

Structure factors: contains datablocks I. DOI: 10.1107/S1600536811016850/bt5538Isup2.hkl

Supplementary material file. DOI: 10.1107/S1600536811016850/bt5538Isup3.cml

Additional supplementary materials:  crystallographic information; 3D view; checkCIF report

## Figures and Tables

**Table 1:** Hydrogen-bond geometry (Å, °)

*D*—H⋯*A*	*D*—H	H⋯*A*	*D*⋯*A*	*D*—H⋯*A*
N1—H1*A*⋯O3	0.89	1.87	2.757 (7)	175
N1—H1*B*⋯O4^i^	0.89	2.04	2.812 (8)	144
N1—H1*C*⋯O5^ii^	0.89	1.91	2.762 (8)	160

Symmetry codes: (i) 
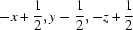
; (ii) 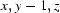.

**Fig. 1.**
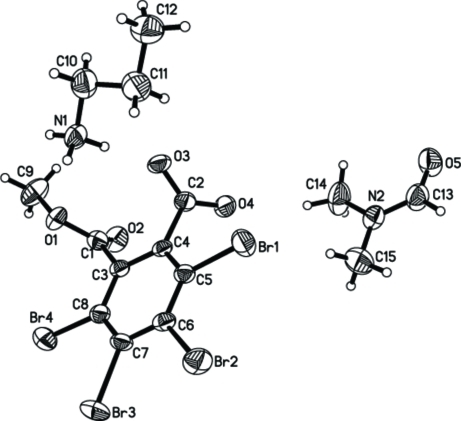
The molecular structure of the title compound drawn with 30% probability ellipsoids.

**Fig. 2.**
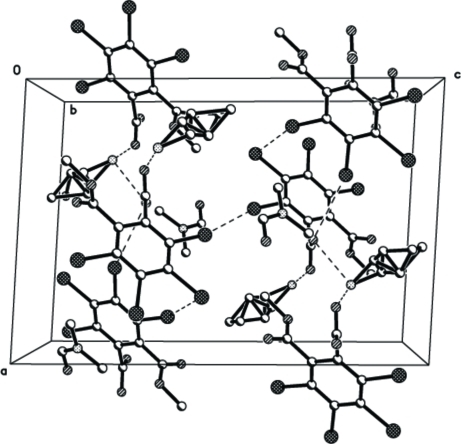
The crystal structure is stabilized by N—H···O hydrogen bonds

## supplementary crystallographic information

### Comment

4,5,6,7-Tetrabromo-2-ethylisoindoline-1,3-dione is an important flame retardant. 3,4,5,6-Tetrabromo-2-(methoxycarbonyl)benzoic acid is an intermediate in the synthesis of this flame retardant. The asymmetric unit of the title compound   contains one propan-1-aminium cation, one 3,4,5,6-tetrabromo-2-(methoxycarbonyl)benzoate anion and one *N*,*N*-dimethylformamide solvent molecule (Fig. 1). In the anion of the title compound, the dihedral angles formed by the benzene ring and the mean planes of the carboxylate and methoxycarbonyl groups are 64.3 (3) and 75.2 (3) °, respectively. The carbon atoms of the propan-1-aminium cation are disordered. The bond lengths and angles are in agreement with those which are related in ethylammonium 2-(methoxycarbonyl)-3,4,5,6-tetrabromobenzoate methanol solvate (Li, 2011*a*) and in 2-Methylanilinium 3,4,5,6-tetrabromo-2-(methoxycarbonyl)benzoate methanol monosolvate(Li, 2011*b*). The crystal structure is stabilized by N—H···O hydrogen bonds (see Fig. 2 and Table 1).

### Experimental

A mixture of 4,5,6,7-tetrabromoisobenzofuran-1,3-dione (4.64 g, 0.01 mol) and methanol (15 ml) was refluxed for 0.5 h. Then propan-1-amine (0.59 g, 0.01 mol) was added to the above solution and mixed for 30 min at room temperature. After filtration, filter cake was disolved in *N*,*N*-dimethylformamide (10 ml), the solution was kept at room temperature for 15 d. Natural evaporation gave colourless single crystals of the title compound, suitable for X-ray analysis.

### Refinement

H atoms were   refined using a riding model with C—H = 0.96–0.97 Å, N—H = 0.89 Å, O—H = 0.82Å and *U*_iso_(H) = 1.2*U*_eq_(C) or 1.5*U*_eq_(O, N, methyl C).

### Crystal data

**Table:**

C_3_H_10_N^+^·C_9_H_3_Br_4_O_4_^−^·C_3_H_7_NO	*F*(000) = 1216
*M**_r_* = 627.97	*D*_x_ = 1.921 Mg m^−^^3^
Monoclinic, *P*2_1_/*n*	Mo *K*α radiation, λ = 0.71073 Å
*a* = 11.8964 (11) Å	Cell parameters from 1875 reflections
*b* = 10.5198 (10) Å	θ = 2.3–20.1°
*c* = 17.3743 (17) Å	µ = 7.44 mm^−^^1^
β = 93.188 (1)°	*T* = 298 K
*V* = 2171.0 (4) Å^3^	Block, colorless
*Z* = 4	0.40 × 0.37 × 0.24 mm

### Data collection

**Table:**

Bruker SMART CCD diffractometer	3821 independent reflections
Radiation source: fine-focus sealed tube	1836 reflections with *I* > 2σ(*I*)
graphite	*R*_int_ = 0.069
φ and ω scans	θ_max_ = 25.0°, θ_min_ = 2.3°
Absorption correction: multi-scan (*SADABS*; Bruker, 1997)	*h* = −14→14
*T*_min_ = 0.155, *T*_max_ = 0.268	*k* = −12→12
10672 measured reflections	*l* = −10→20

### Refinement

**Table:**

Refinement on *F*^2^	Primary atom site location: structure-invariant direct methods
Least-squares matrix: full	Secondary atom site location: difference Fourier map
*R*[*F*^2^ > 2σ(*F*^2^)] = 0.047	Hydrogen site location: inferred from neighbouring sites
*wR*(*F*^2^) = 0.104	H-atom parameters constrained
*S* = 1.03	*w* = 1/[σ^2^(*F*_o_^2^) + (0.0346*P*)^2^] where *P* = (*F*_o_^2^ + 2*F*_c_^2^)/3
3821 reflections	(Δ/σ)_max_ = 0.001
268 parameters	Δρ_max_ = 0.57 e Å^−^^3^
0 restraints	Δρ_min_ = −0.38 e Å^−^^3^

### Special details

**Table:**

Geometry. All e.s.d.'s (except the e.s.d. in the dihedral angle between two l.s. planes) are estimated using the full covariance matrix. The cell e.s.d.'s are taken into account individually in the estimation of e.s.d.'s in distances, angles and torsion angles; correlations between e.s.d.'s in cell parameters are only used when they are defined by crystal symmetry. An approximate (isotropic) treatment of cell e.s.d.'s is used for estimating e.s.d.'s involving l.s. planes.
Refinement. Refinement of *F*^2^ against ALL reflections. The weighted *R*-factor *wR* and goodness of fit *S* are based on *F*^2^, conventional *R*-factors *R* are based on *F*, with *F* set to zero for negative *F*^2^. The threshold expression of *F*^2^ > σ(*F*^2^) is used only for calculating *R*-factors(gt) *etc*. and is not relevant to the choice of reflections for refinement. *R*-factors based on *F*^2^ are statistically about twice as large as those based on *F*, and *R*- factors based on ALL data will be even larger.

### Fractional atomic coordinates and isotropic or equivalent isotropic displacement parameters (Å^2^)

**Table:**

	*x*	*y*	*z*	*U*_iso_*/*U*_eq_	Occ. (<1)
Br1	0.54990 (6)	0.36358 (7)	0.43979 (5)	0.0768 (3)	
Br2	0.77382 (6)	0.18088 (8)	0.44525 (5)	0.0787 (3)	
Br3	0.82232 (6)	−0.00007 (9)	0.29589 (6)	0.0923 (3)	
Br4	0.63517 (6)	−0.01203 (8)	0.14923 (5)	0.0828 (3)	
N1	0.2391 (5)	0.0691 (6)	0.3225 (4)	0.0701 (18)	
H1A	0.2650	0.1457	0.3109	0.105*	0.65 (3)
H1B	0.2129	0.0305	0.2796	0.105*	0.65 (3)
H1C	0.2947	0.0231	0.3447	0.105*	0.65 (3)
H1'A	0.1918	0.0366	0.2862	0.105*	0.35 (3)
H1'B	0.3018	0.0227	0.3261	0.105*	0.35 (3)
H1'C	0.2560	0.1487	0.3102	0.105*	0.35 (3)
N2	0.5289 (5)	0.8141 (7)	0.3665 (4)	0.0697 (18)	
O1	0.3696 (4)	0.0717 (5)	0.1644 (3)	0.0631 (14)	
O2	0.4289 (4)	0.2556 (5)	0.1162 (3)	0.0776 (16)	
O3	0.3100 (3)	0.3064 (5)	0.2784 (3)	0.0721 (15)	
O4	0.4291 (4)	0.4662 (5)	0.2792 (3)	0.0740 (16)	
O5	0.4306 (5)	0.9836 (5)	0.4037 (3)	0.0917 (19)	
C1	0.4351 (6)	0.1731 (8)	0.1638 (4)	0.056 (2)	
C2	0.4049 (6)	0.3538 (7)	0.2822 (4)	0.0541 (19)	
C3	0.5206 (5)	0.1754 (6)	0.2302 (4)	0.0438 (17)	
C4	0.5034 (5)	0.2591 (6)	0.2915 (4)	0.0432 (17)	
C5	0.5786 (5)	0.2591 (6)	0.3546 (4)	0.0471 (18)	
C6	0.6718 (5)	0.1788 (7)	0.3581 (4)	0.0501 (18)	
C7	0.6908 (5)	0.1006 (6)	0.2960 (4)	0.0522 (19)	
C8	0.6138 (5)	0.0966 (6)	0.2343 (4)	0.0514 (19)	
C9	0.2842 (6)	0.0614 (8)	0.1018 (4)	0.092 (3)	
H9A	0.2345	0.1332	0.1029	0.138*	
H9B	0.2420	−0.0154	0.1075	0.138*	
H9C	0.3197	0.0597	0.0535	0.138*	
C10	0.146 (3)	0.082 (4)	0.377 (3)	0.092 (10)	0.65 (3)
H10A	0.1269	0.0002	0.3975	0.110*	0.65 (3)
H10B	0.0798	0.1186	0.3504	0.110*	0.65 (3)
C11	0.1904 (17)	0.169 (2)	0.4401 (13)	0.105 (8)	0.65 (3)
H11A	0.2540	0.1306	0.4687	0.125*	0.65 (3)
H11B	0.2141	0.2496	0.4192	0.125*	0.65 (3)
C12	0.0942 (16)	0.188 (3)	0.4911 (12)	0.109 (7)	0.65 (3)
H12A	0.0279	0.2109	0.4602	0.164*	0.65 (3)
H12B	0.1124	0.2546	0.5274	0.164*	0.65 (3)
H12C	0.0806	0.1106	0.5183	0.164*	0.65 (3)
C10'	0.191 (5)	0.079 (9)	0.398 (5)	0.091 (18)	0.35 (3)
H10C	0.2502	0.0933	0.4382	0.109*	0.35 (3)
H10D	0.1521	0.0006	0.4103	0.109*	0.35 (3)
C11'	0.110 (3)	0.190 (4)	0.394 (2)	0.099 (14)	0.35 (3)
H11C	0.1282	0.2414	0.3501	0.119*	0.35 (3)
H11D	0.0349	0.1553	0.3821	0.119*	0.35 (3)
C12'	0.103 (4)	0.275 (4)	0.461 (4)	0.120 (16)	0.35 (3)
H12D	0.1432	0.2385	0.5045	0.180*	0.35 (3)
H12E	0.0251	0.2863	0.4720	0.180*	0.35 (3)
H12F	0.1348	0.3560	0.4490	0.180*	0.35 (3)
C13	0.5086 (7)	0.9088 (9)	0.4133 (5)	0.084 (3)	
H13	0.5571	0.9195	0.4567	0.101*	
C14	0.4573 (7)	0.7909 (8)	0.2986 (5)	0.111 (3)	
H14A	0.3816	0.8159	0.3080	0.166*	
H14B	0.4587	0.7021	0.2862	0.166*	
H14C	0.4837	0.8393	0.2564	0.166*	
C15	0.6236 (7)	0.7288 (8)	0.3804 (6)	0.113 (3)	
H15A	0.6662	0.7546	0.4263	0.169*	
H15B	0.6709	0.7317	0.3374	0.169*	
H15C	0.5966	0.6437	0.3868	0.169*	

### Atomic displacement parameters (Å^2^)

**Table:**

	*U*^11^	*U*^22^	*U*^33^	*U*^12^	*U*^13^	*U*^23^
Br1	0.0844 (6)	0.0688 (6)	0.0766 (6)	0.0133 (4)	−0.0017 (5)	−0.0155 (5)
Br2	0.0667 (5)	0.0806 (6)	0.0862 (7)	0.0101 (4)	−0.0205 (5)	0.0123 (5)
Br3	0.0556 (4)	0.0903 (7)	0.1302 (8)	0.0342 (5)	−0.0014 (5)	−0.0098 (6)
Br4	0.0749 (5)	0.0825 (6)	0.0923 (7)	0.0100 (5)	0.0163 (5)	−0.0266 (5)
N1	0.060 (4)	0.068 (4)	0.080 (5)	−0.008 (3)	−0.011 (4)	−0.011 (4)
N2	0.071 (4)	0.061 (4)	0.076 (5)	−0.011 (4)	−0.002 (4)	0.001 (4)
O1	0.057 (3)	0.063 (3)	0.068 (4)	−0.018 (3)	−0.009 (3)	0.002 (3)
O2	0.085 (4)	0.073 (4)	0.073 (4)	−0.007 (3)	−0.009 (3)	0.018 (3)
O3	0.034 (3)	0.057 (3)	0.124 (5)	0.003 (2)	−0.001 (3)	0.008 (3)
O4	0.055 (3)	0.038 (3)	0.128 (5)	0.003 (2)	−0.001 (3)	0.008 (3)
O5	0.098 (4)	0.076 (4)	0.097 (5)	0.026 (3)	−0.027 (4)	−0.012 (4)
C1	0.054 (4)	0.056 (6)	0.059 (6)	0.006 (4)	0.007 (4)	0.004 (5)
C2	0.045 (4)	0.054 (5)	0.062 (5)	0.007 (4)	−0.006 (4)	0.004 (4)
C3	0.035 (4)	0.044 (4)	0.053 (5)	−0.002 (3)	0.003 (4)	0.009 (4)
C4	0.030 (3)	0.042 (4)	0.057 (5)	−0.004 (3)	0.005 (4)	0.010 (4)
C5	0.039 (4)	0.045 (4)	0.058 (5)	0.002 (3)	0.001 (4)	0.006 (4)
C6	0.037 (4)	0.051 (4)	0.063 (5)	−0.002 (3)	0.004 (4)	0.014 (4)
C7	0.037 (4)	0.048 (5)	0.072 (6)	0.011 (3)	0.006 (4)	0.008 (4)
C8	0.042 (4)	0.041 (4)	0.071 (5)	0.000 (3)	0.007 (4)	0.010 (4)
C9	0.081 (5)	0.125 (7)	0.065 (6)	−0.034 (5)	−0.025 (5)	0.002 (6)
C10	0.09 (2)	0.078 (17)	0.11 (3)	0.005 (19)	−0.018 (18)	−0.008 (16)
C11	0.086 (13)	0.107 (17)	0.119 (17)	0.008 (11)	−0.011 (13)	−0.025 (14)
C12	0.099 (12)	0.12 (2)	0.107 (15)	−0.005 (15)	0.028 (11)	−0.008 (15)
C10'	0.08 (4)	0.09 (3)	0.10 (6)	0.01 (3)	−0.02 (3)	−0.01 (3)
C11'	0.08 (2)	0.11 (3)	0.11 (3)	0.01 (2)	−0.01 (2)	−0.01 (3)
C12'	0.12 (3)	0.08 (3)	0.15 (4)	0.01 (2)	0.00 (3)	−0.03 (3)
C13	0.083 (6)	0.078 (7)	0.088 (7)	0.002 (5)	−0.021 (6)	−0.011 (6)
C14	0.134 (8)	0.086 (7)	0.106 (8)	0.000 (6)	−0.046 (7)	−0.017 (6)
C15	0.096 (6)	0.093 (7)	0.149 (10)	0.032 (6)	−0.004 (6)	−0.011 (7)

### Geometric parameters (Å, °)

**Table:**

Br1—C5	1.890 (7)	C9—H9A	0.9600
Br2—C6	1.887 (6)	C9—H9B	0.9600
Br3—C7	1.889 (6)	C9—H9C	0.9600
Br4—C8	1.897 (7)	C10—C11	1.50 (6)
N1—C10'	1.47 (10)	C10—H10A	0.9700
N1—C10	1.50 (5)	C10—H10B	0.9700
N1—H1A	0.8900	C11—C12	1.50 (2)
N1—H1B	0.8900	C11—H11A	0.9700
N1—H1C	0.8900	C11—H11B	0.9700
N1—H1'A	0.8900	C12—H12A	0.9600
N1—H1'B	0.8900	C12—H12B	0.9600
N1—H1'C	0.8900	C12—H12C	0.9600
N2—C13	1.318 (9)	C10'—C11'	1.52 (10)
N2—C14	1.436 (8)	C10'—H10C	0.9700
N2—C15	1.450 (9)	C10'—H10D	0.9700
O1—C1	1.322 (8)	C11'—C12'	1.47 (5)
O1—C9	1.451 (7)	C11'—H11C	0.9700
O2—C1	1.198 (7)	C11'—H11D	0.9700
O3—C2	1.232 (7)	C12'—H12D	0.9600
O4—C2	1.220 (7)	C12'—H12E	0.9600
O5—C13	1.221 (9)	C12'—H12F	0.9600
C1—C3	1.495 (9)	C13—H13	0.9300
C2—C4	1.539 (8)	C14—H14A	0.9600
C3—C8	1.383 (8)	C14—H14B	0.9600
C3—C4	1.406 (8)	C14—H14C	0.9600
C4—C5	1.375 (8)	C15—H15A	0.9600
C5—C6	1.393 (8)	C15—H15B	0.9600
C6—C7	1.386 (9)	C15—H15C	0.9600
C7—C8	1.371 (8)		
			
C10'—N1—C10	24.7 (18)	C7—C8—Br4	121.0 (5)
C10'—N1—H1A	107.3	C3—C8—Br4	117.5 (5)
C10—N1—H1A	109.5	O1—C9—H9A	109.5
C10'—N1—H1B	130.3	O1—C9—H9B	109.5
C10—N1—H1B	109.5	H9A—C9—H9B	109.5
H1A—N1—H1B	109.5	O1—C9—H9C	109.5
C10'—N1—H1C	87.9	H9A—C9—H9C	109.5
C10—N1—H1C	109.5	H9B—C9—H9C	109.5
H1A—N1—H1C	109.5	N1—C10—C11	106 (2)
H1B—N1—H1C	109.5	N1—C10—H10A	110.5
C10'—N1—H1'A	113.6	C11—C10—H10A	110.5
C10—N1—H1'A	91.3	N1—C10—H10B	110.5
H1A—N1—H1'A	113.6	C11—C10—H10B	110.5
H1B—N1—H1'A	18.7	H10A—C10—H10B	108.7
H1C—N1—H1'A	121.6	C12—C11—C10	105 (2)
C10'—N1—H1'B	109.9	C12—C11—H11A	110.6
C10—N1—H1'B	130.6	C10—C11—H11A	110.6
H1A—N1—H1'B	102.4	C12—C11—H11B	110.6
H1B—N1—H1'B	93.6	C10—C11—H11B	110.6
H1C—N1—H1'B	22.0	H11A—C11—H11B	108.8
H1'A—N1—H1'B	109.5	N1—C10'—C11'	106 (5)
C10'—N1—H1'C	104.9	N1—C10'—H10C	110.4
C10—N1—H1'C	104.3	C11'—C10'—H10C	110.4
H1A—N1—H1'C	7.2	N1—C10'—H10D	110.4
H1B—N1—H1'C	107.5	C11'—C10'—H10D	110.4
H1C—N1—H1'C	116.4	H10C—C10'—H10D	108.6
H1'A—N1—H1'C	109.5	C12'—C11'—C10'	119 (4)
H1'B—N1—H1'C	109.5	C12'—C11'—H11C	107.5
C13—N2—C14	121.0 (7)	C10'—C11'—H11C	107.5
C13—N2—C15	122.2 (8)	C12'—C11'—H11D	107.5
C14—N2—C15	116.9 (7)	C10'—C11'—H11D	107.5
C1—O1—C9	116.2 (6)	H11C—C11'—H11D	107.0
O2—C1—O1	125.3 (7)	C11'—C12'—H12D	109.5
O2—C1—C3	122.2 (7)	C11'—C12'—H12E	109.5
O1—C1—C3	112.5 (7)	H12D—C12'—H12E	109.5
O4—C2—O3	127.5 (6)	C11'—C12'—H12F	109.5
O4—C2—C4	116.8 (6)	H12D—C12'—H12F	109.5
O3—C2—C4	115.7 (6)	H12E—C12'—H12F	109.5
C8—C3—C4	119.1 (6)	O5—C13—N2	124.5 (8)
C8—C3—C1	122.7 (6)	O5—C13—H13	117.7
C4—C3—C1	118.2 (6)	N2—C13—H13	117.7
C5—C4—C3	119.2 (6)	N2—C14—H14A	109.5
C5—C4—C2	122.8 (6)	N2—C14—H14B	109.5
C3—C4—C2	117.9 (6)	H14A—C14—H14B	109.5
C4—C5—C6	121.1 (6)	N2—C14—H14C	109.5
C4—C5—Br1	119.1 (5)	H14A—C14—H14C	109.5
C6—C5—Br1	119.7 (5)	H14B—C14—H14C	109.5
C7—C6—C5	119.3 (6)	N2—C15—H15A	109.5
C7—C6—Br2	120.4 (5)	N2—C15—H15B	109.5
C5—C6—Br2	120.2 (6)	H15A—C15—H15B	109.5
C8—C7—C6	119.7 (6)	N2—C15—H15C	109.5
C8—C7—Br3	120.0 (6)	H15A—C15—H15C	109.5
C6—C7—Br3	120.3 (5)	H15B—C15—H15C	109.5
C7—C8—C3	121.4 (6)		
			
C9—O1—C1—O2	0.1 (10)	C4—C5—C6—Br2	179.9 (5)
C9—O1—C1—C3	−179.8 (6)	Br1—C5—C6—Br2	−3.4 (7)
O2—C1—C3—C8	−106.3 (8)	C5—C6—C7—C8	−4.5 (10)
O1—C1—C3—C8	73.6 (8)	Br2—C6—C7—C8	177.6 (5)
O2—C1—C3—C4	75.4 (9)	C5—C6—C7—Br3	175.3 (5)
O1—C1—C3—C4	−104.8 (7)	Br2—C6—C7—Br3	−2.6 (8)
C8—C3—C4—C5	−1.0 (9)	C6—C7—C8—C3	4.3 (10)
C1—C3—C4—C5	177.4 (6)	Br3—C7—C8—C3	−175.5 (5)
C8—C3—C4—C2	174.8 (6)	C6—C7—C8—Br4	−178.6 (5)
C1—C3—C4—C2	−6.9 (9)	Br3—C7—C8—Br4	1.5 (7)
O4—C2—C4—C5	61.8 (9)	C4—C3—C8—C7	−1.5 (9)
O3—C2—C4—C5	−118.4 (7)	C1—C3—C8—C7	−179.9 (6)
O4—C2—C4—C3	−113.8 (7)	C4—C3—C8—Br4	−178.7 (4)
O3—C2—C4—C3	65.9 (9)	C1—C3—C8—Br4	3.0 (8)
C3—C4—C5—C6	0.8 (9)	C10'—N1—C10—C11	40 (10)
C2—C4—C5—C6	−174.8 (6)	N1—C10—C11—C12	176 (2)
C3—C4—C5—Br1	−175.9 (4)	C10—N1—C10'—C11'	−47 (9)
C2—C4—C5—Br1	8.6 (8)	N1—C10'—C11'—C12'	−141 (4)
C4—C5—C6—C7	2.0 (9)	C14—N2—C13—O5	−0.5 (13)
Br1—C5—C6—C7	178.6 (5)	C15—N2—C13—O5	179.4 (8)

### Hydrogen-bond geometry (Å, °)

**Table:**

*D*—H···*A*	*D*—H	H···*A*	*D*···*A*	*D*—H···*A*
N1—H1A···O3	0.89	1.87	2.757 (7)	175
N1—H1B···O4^i^	0.89	2.04	2.812 (8)	144
N1—H1C···O5^ii^	0.89	1.91	2.762 (8)	160

Symmetry codes: (i) −*x*+1/2, *y*−1/2, −*z*+1/2; (ii) *x*, *y*−1, *z*.
